# An innovative approach for determining the customized refractive index of ectatic corneas in cataractous patients

**DOI:** 10.1038/s41598-020-73492-4

**Published:** 2020-10-07

**Authors:** Shiva Pirhadi, Keivan Maghooli, Khosrow Jadidi

**Affiliations:** 1 Department of Biomedical Engineering, Science and Research Branch, Islamic Azad University, Tehran, Iran; 2 Vision Health Research Center, Semnan University of Medical Sciences, Semnan, Iran

**Keywords:** Diseases, Eye diseases, Corneal diseases

## Abstract

The aim of this study is to determine the customized refractive index of ectatic corneas and also propose a method for determining the corneal and IOL power in these eyes. Seven eyes with moderate and severe corneal ectatic disorders, which had been under cataract surgery, were included. At least three months after cataract surgery, axial length, cornea, IOL thickness and the distance between IOL from cornea, and aberrometry were measured. All the measured points of the posterior and anterior parts of the cornea converted to points cloud and surface by using the MATLAB and Solidworks software. The implanted IOLs were designed by Zemax software. The ray tracing analysis was performed on the customized eye models, and the corneal refractive index was determined by minimizing the difference between the measured aberrations from the device and resulted aberrations from the simulation. Then, by the use of preoperative corneal images, corneal power was calculated by considering the anterior and posterior parts of the cornea and refractive index of 1.376 and the customized corneal refractive index in different regions and finally it was entered into the IOL power calculation formulas. The corneal power in the 4 mm region and the Barrett formula resulted the prediction error of six eyes within ± 1 diopter. It seems that using the total corneal power along with the Barrett formula can prevent postoperative hyperopic shift, especially in eyes with advanced ectatic disorders.

## Introduction

Keratoconus (KCN) and Pellucid marginal degeneration (PMD) are non-inflammatory and almost progressive corneal diseases known by ectasia, central, paracentral and peripheral corneal thinning^1^. These changes result in irregular Astigmatism and reduced vision. Causes of these problems, stopping the progress of the disease and improving the vision of patients consider as a challenging task. A study on more than 4 million patients aged 10–40 years old showed that the incidence of keratoconus was 1:7500 in the relevant age category and its estimated prevalence in the general population was 1:375. These results were fivefold to tenfold higher than previously reported^2^. Although there is no evidence of the incidence or prevalence of PMD, studies have shown that its outbreak is less than keratoconus^3^.

With regards to the population growth rate of people aged above 65 years old, which is reported to be 15.1%, the number of patients with cataract will increase^4^. On the other hand, keratoconus patients are more likely to get cataract than non-keratoconus patients^5^. Therefore, it is necessary for ophthalmologists to prepare for a growing population of patients with keratoconus or PMD that require cataract surgery. Patients with advanced keratoconus may be candidates for keratoplasty with cataract extraction and intraocular lens implantation (IOL) but patients who have had a well-corrected vision with contact lenses or glasses before cataract may not require corneal surgery^6^. For these people, the accurate IOL power calculation is a challenge, while determining the correct IOL power plays an important role in achieving acceptable visual results and optimal refractive and also in patients satisfaction after cataract surgery. The IOL power calculation requires three basic factors: corneal power measurement, effective lens positioning (ELP) and axial length measurements that in these patients due to increase in eye length, irregular corneal surface and advanced astigmatism, an accurate IOL power with high reliability is not being calculated^7^.

As discussed earlier, one of the factors of IOL power calculation is to determine the corneal refractive power. Corneal power accounts for about 2/3 of the total dioptric power of ocular refractive system. An incorrect corneal power will cause errors in the various parts of IOL power calculation. Unfortunately, corneal power is not calculated through a straight process and no keratometry device directly measures corneal power^8^. In fact, many computerized videokeratography, keratometry and biometry devices estimate the total dioptric power of the cornea in terms of diopter by determining the radius of the anterior corneal curvature and by the use of an indicator called corneal refractive index or keratometric index (1.3375)^9^. This value is based on convenience and results in a value of 7.5 mm for the anterior corneal radius of curvature corresponding to a corneal power of exactly 45D^10^. Although this index provides great results in virgin normal eyes IOL power calculation, it can change in surgeries or disease processes.

If the accepted assumptions for normal eyes enter into biometric calculations of the eyes with corneal ectasia, the power of the cornea usually will be overestimated and consequently the IOL power will be underestimated that leads to hyperopia in patients after surgery^6,11,12^. Therefore, if the corneal power calculation of these patients is performed only based on the anterior curvature and standard corneal index, an accurate IOL power cannot be achieved. In order to determine the refractive power of the cornea, anterior segment, thickness and exact corneal refractive index that may have changed with the disease process must be considered. In this study, we tend to examine this hypothesis that the corneal refractive index may change with diseases related to corneal ectasia. According to our knowledge, no research has been done to investigate the corneal refractive index of patients with KCN and PMD. For the first time, this study aims to calculate the refractive index of such corneas in moderate and severe stages of the disease. For this purpose, we simulated eyes, which had been under cataract surgery, and their corneas diagnosed with KCN or PMD. By the use of Ray Tracing method, we tried to calculate individuals’ corneal refractive index in its customized model of each patient eye. In addition, we plan to find out the effect of calculating the total corneal power in determining the more accurate IOL power.

## Results

In this study, 7 eyes of 5 patients with PMD or KCN were evaluated. Six eyes with KCN were categorized into moderate and severe groups based on Amsler-Krumeich classification (Based on this classification, the average keratometric readings of ≤ 48 D is considered as mild, > 48 D and ≤ 55 diopters is considered as moderate, and > 55 is considered as severe^13^). These eyes had been under phacoemulsification surgery and intraocular lens implantation. Demographic information, ectasia type, keratometry (measured by Pentacam), and biometry values before each patient's surgery are shown in Table 1. In addition, Table 2 shows the type and power of the implanted IOL and the subjective refraction of each patient measured at least 3 months after surgery.

**Table 1:** Demographic information, corneal ectasia type, keratometry and biometry of the patients.

Subject	Eye	Gender	Age (years)	Type of ectasia	K1 (D)	K2 (D)	Mean K (D)	AL (mm)	ACD (mm)	LT (mm)	WTW (mm)
S1	OD	Female	65	PMD	40.6	50.2	45.4	23.12	3.37	–	–
S2	OD	Male	42	KCN	52.9	54.6	53.75	30.08	3.67	4.81	12.2
S2	OS	Male	42	KCN	53.7	56.9	55.3	30.67	3.83	4.14	12.0
S3	OS	Female	63	Keratoglobus	53.3	59.5	56.4	23.34	3.99	3.44	11.8
S4	OS	Female	77	KCN	49.4	52.6	51.0	25.82	3.34	4.14	11.4
S5	OD	Female	63	KCN	48.6	52.6	50.6	24.00	3.38	3.83	10.9
S5	OS	Female	63	KCN	53.4	56.0	54.7	23.77	3.44	3.83	11.8

**Table 2:** Type and power of the implanted IOLs and refraction of the patients after surgery.

Subject	Eye	IOL type	Name of IOL	Model	IOL power (D)	IOL cylinder power (D)	Postoperative sphere (D)	Postoperative cylinder (D)
S1	OD	Toric	Acrysof IQ	SN6AT9	+ 22.0	6	+ 2.5	− 6.0
S2	OD	Non-toric	Ophthalight	ML FLEX 2AS	− 8.0	–	0.0	− 3.0
S2	OS	Non-toric	Ophthalight	ML FLEX 2AS	− 9.0	–	− 1.0	− 6.0
S3	OS	Non-toric	Envista	MX60	0.0	–	+ 4.5	− 5.5
S4	OS	Non-toric	Envista	MX60	+ 7.0	–	0.0	− 3.0
S5	OD	Non-toric	Tecnis	ZCB00	+ 11.0	–	+ 1.0	− 4.0
S5	OS	Non-toric	PreciSAL	302AC	+ 4.0	–	+ 3.0	− 3.0

### Determining the eyes customized refractive index

After designing the intraocular lens and creating a customized model for each patient’s eye, the ray tracing method was implemented by using optical analysis software. The aberrations of each eye were calculated using this method. Then by applying the optimization process, we tried to minimize the difference between the aberrations of simulation and those measured from the device. Table 3 demonstrates the absolute value of the difference between simulated aberrations and measured ones in different eyes for different Zernike coefficients. By minimizing the difference between aberrations, the unknown parameter (i.e., refractive index of the cornea), was calculated by the optimization method. These two parameters were specifically obtained for the studied eyes, which are shown in Table 4. As you can see, the refractive indexes of the ectatic corneas deviated from the standard value (1.376), so that the refractive index increased in four of seven eyes. According to the small number of samples, it cannot be stated with certainty that the refractive index in these corneas is higher than the standard corneal refractive index. But it seems that increased stress and strain caused by ectasia can lead to more tightly packed collagen lamellae and changes in refractive index^14^.

**Table 3:** The absolute value of the difference between simulated aberrations and measured ones by the aberrometer for 18 different Zernike coefficients and the seven studied eyes.

Aberration	Zernike term Z_n_^m^	The absolute difference between measured aberrations and simulated aberrations for subjects						
		S1 OD	S2 OD	S2 OS	S3 OS	S4 OS	S5 OD	S5 OS
Oblique astigmatism	Z_2_^−2^	0.125	0.035	0.343	0.052	0.256	0.116	0.055
Defocus	Z_2_^0^	0.075	0.115	0.107	0.027	0.009	0.132	0.115
Vertical astigmatism	Z_2_^2^	0.227	0.606	0.167	0.106	0.337	0.374	0.015
Vertical trefoil	Z_3_^−3^	0.151	0.425	0.055	0.065	0.060	0.155	0.086
Vertical coma	Z_3_^−1^	0.091	0.128	0.282	0.028	0.139	0.268	0.162
Horizontal coma	Z_3_^1^	0.083	0.249	0.090	0.100	0.021	0.282	0.388
Oblique trefoil	Z_3_^3^	0.044	0.335	0.177	0.110	0.350	0.165	0.216
Oblique quadrafoil	Z_4_^−4^	0.028	0.001	0.095	0.072	0.023	0.031	0.044
Oblique secondary astigmatism	Z_4_^−2^	0.004	0.045	0.012	0.026	0.079	0.005	0.065
Primary spherical	Z_4_^0^	0.018	0.001	0.138	0.018	0.062	0.017	0.016
Vertical Secondary Astigmatism	Z_4_^2^	0.018	0.306	0.098	0.037	0.096	0.024	0.208
Vertical quadrafoil	Z_4_^4^	0.039	0.048	0.028	0.012	0.259	0.010	0.018
Vertical pentafoil	Z_5_^−5^	0.018	0.004	0.114	0.028	0.142	0.024	0.019
Vertical secondary trefoil	Z_5_^−3^	0.021	0.104	0.006	0.012	0.047	0.029	0.012
Vertical secondary coma	Z_5_^−1^	0.004	0.005	0.242	0.001	0.044	0.012	0.009
Horizontal secondary coma	Z_5_^1^	0.002	0.013	0.077	0.029	0.052	0.038	0.117
Oblique secondary trefoil	Z_5_^3^	0.007	0.039	0.052	0.021	0.094	0.011	0.110
Oblique pentafoil	Z_5_^5^	0.026	0.012	0.080	0.013	0.004	0.015	0.001

**Table 4:** The results of optimization customized refractive index of corneas.

Parameter	Subject						
	S1-OD	S2-OD	S2-OS	S3-OS	S4-OS	S5-OD	S5-OS
Corneal refractive index	1.383	1.388	1.365	1.381	1.403	1.372	1.371

### Determine the corneal power and IOL power of the eyes

The corneal power of each eye was calculated by the use of keratometry values measured by IOLMaster and keratometry values obtained from the customized keratometric index. Moreover, corneal power was calculated by the use of our proposed method in different areas of 0.5, 1, 2, 3 and 4 mm for normal and customized refractive index of each eye. The corneal power values from different methods were entered into the computational formulas for determining the power of the IOL.

For the power of implanted IOL for patients, the predicted refraction value of each formula was determined. Then, the PE was calculated based on the refraction after surgery and the predicted refraction of each formula. Table 5 provides PE values for different values of corneal power and different formulas. As you can see, the calculated keratometry values with the customized index have fewer errors than the index of 1.3375. However, the minimum error is related to the calculated corneal power based on our proposed method in the 4 mm region by applying the Barrett formula. The calculated power values based on the proposed methodology, led to have six eyes out of seven with the refractive PEs in the range of ± 0.5 D.

**Table 5:** The prediction error of IOL power calculation formulas based on different values of corneal power.

Subject	Formula	Corneal power using S.KI	Corneal power using I.KI	Corneal calculated power based on our proposed method									
				0.5 mm		1.0 mm		2.0 mm		3.0 mm		4.0 mm	
				N.C.I	I.C.I	N.C.I	I.C.I	N.C.I	I.C.I	N.C.I	I.C.I	N.C.I	I.C.I
S1-OD	SRK/T	2.58	1.96	1.68	1.54	1.73	1.58	1.89	1.76	2.07	1.95	2.27	2.16
	Holladay2	2.91	2.18	1.78	1.63	1.84	1.69	2.05	1.90	2.26	2.12	2.48	2.35
	HofferQ	2.48	1.71	1.35	1.18	1.41	1.24	1.62	1.46	1.84	1.69	2.09	1.95
	Haigis	2.42	1.58	1.20	1.02	1.27	1.08	1.49	1.31	1.73	1.57	2.00	1.84
	Barrett	2.93	2.20	1.81	1.66	1.87	1.72	2.07	1.93	2.28	2.15	2.50	2.37
	Barrett toric	− 0.03	− 0.57	− 0.49	− 0.64	− 0.49	− 0.63	− 0.39	− 0.53	− 0.25	− 0.35	**0.0**	− 0.12
S2-OD	SRK/T	6.16	3.49	6.40	5.51	6.09	5.23	5.09	4.28	3.63	2.91	2.08	1.48
	Holladay2	6.29	3.48	6.52	5.60	6.18	5.29	5.13	4.29	3.61	2.85	1.98	1.34
	HofferQ	6.89	3.85	7.16	6.14	6.80	5.83	5.66	4.75	4.02	3.21	2.28	1.60
	Haigis	4.57	1.88	4.81	3.92	4.50	3.64	3.49	2.68	2.03	1.30	0.47	− 0.14
	Barrett	4.1	1.3	4.32	3.41	3.99	3.10	2.94	2.11	1.43	0.68	− **0.19**	− 0.81
S2-OS	SRK/T	5.96	3.51	5.88	6.45	5.84	6.42	5.18	5.76	4.18	4.72	3.04	3.55
	Holladay2	6.17	3.57	6.03	6.63	5.99	6.60	5.31	5.91	4.25	4.81	3.05	3.58
	HofferQ	6.81	3.98	6.71	7.38	6.67	7.34	5.91	6.57	4.75	5.37	3.44	4.03
	Haigis	4.30	1.82	4.21	4.79	4.17	4.76	3.51	4.09	2.50	3.05	1.35	1.86
	Barrett	3.92	1.33	3.78	4.38	3.74	4.34	3.06	3.66	2.01	2.57	**0.81**	1.34
S3-OS	SRK/T	5.30	2.95	4.62	4.27	4.43	4.07	3.50	3.17	2.31	2.04	1.19	0.95
	Holladay2	5.68	3.29	4.97	4.60	4.77	4.40	3.81	3.47	2.58	6.71	1.41	1.17
	HofferQ	5.64	3.24	4.95	4.59	4.75	4.38	3.80	3.47	2.59	2.31	1.45	1.20
	Haigis	4.59	2.28	3.92	3.58	3.73	3.38	2.83	2.50	1.66	2.51	0.56	0.33
	Barrett	NC	1.68	NC	NC	NC	NC	NC	1.88	1.02	0.75	− **0.09**	− 0.33
S4-OS	SRK/T	3.29	0.54	− 0.42	− 1.61	− 0.13	− 1.35	− 0.01	− 1.15	− 0.40	− 1.23	− 0.69	− 1.24
	Holladay2	4.35	1.65	0.71	− 1.07	0.99	− 0.74	1.11	− 0.45	0.76	− 0.57	0.49	− 0.57
	HofferQ	4.47	1.84	0.92	− 0.91	1.21	− 0.57	1.32	− 0.29	0.95	− 0.41	0.67	− 0.41
	Haigis	4.22	1.54	0.61	− 1.24	0.90	− 0.90	1.01	− 0.61	0.64	− 0.74	0.35	− 0.74
	Barrett	4.04	1.43	0.48	− 1.30	0.77	− 0.97	0.89	− 0.68	0.54	− 0.80	**0.26**	− 0.80
S5-OD	SRK/T	− 0.31	− 1.17	− 0.32	− 0.32	− 0.36	− 0.31	− 0.53	− 0.43	− 0.72	− 0.62	− 0.95	− 0.87
	Holladay2	1.45	− 0.49	1.01	1.17	0.86	1.01	0.50	0.64	0.17	0.29	− 0.19	− 0.11
	HofferQ	1.63	− 0.33	1.15	1.34	1.01	1.20	0.65	0.83	0.33	0.48	− 0.03	0.09
	Haigis	0.43	− 0.62	0.93	1.13	0.78	0.98	0.41	0.60	0.07	0.24	− 0.30	− 0.18
	Barrett	1.29	− 0.64	0.87	1.07	0.72	0.91	0.36	0.54	0.03	0.19	− **0.33**	− 0.22
S5-OS	SRK/T	5.87	3.04	5.63	6.05	5.36	5.74	4.30	4.66	2.87	3.19	1.36	1.61
	Holladay2	6.52	3.71	6.21	6.64	5.94	6.35	4.90	5.27	3.48	3.82	1.98	2.24
	HofferQ	6.47	3.74	6.25	6.64	5.98	6.35	4.96	5.30	3.58	3.89	2.11	3.65
	Haigis	5.92	3.16	5.69	6.09	5.42	5.80	4.39	4.74	3.00	3.31	1.53	1.77
	Barrett	4.80	2.15	4.52	4.90	4.26	4.62	3.28	3.61	1.94	2.24	**0.53**	0.75

Bold numbers indicate the lowest PE value for all eyes for the most appropriate corneal power and IOL calculation formula.

S.KI: standard keratometry index (1.3375); I.KI: individual corneal keratometry index; N.C.I: normal corneal index (1.376); I.C.I: individual corneal index; NC: not calculable. The Barrett formula for our third patient’s left eye was not applicable since his calculated values of corneal power were more than 60 diopters.

## Discussion

Although the number of eyes entered into this study is not high, based on to our best knowledge, this is the first study that focuses on determining the refractive index of the cornea in patients with keratoconus. Moreover, this is the only study that tries to evaluate the ray tracing technique and finding a method for determining the corneal power of these patients in order to obtain a suitable IOL power. In previous studies, it has been stated that choosing the IOL power for keratoconus patients is a challenge, especially in patients with corneal keratometry of more than 48 diopters^11,13^. Therefore, only the moderate and severe keratoconus corneas were studied in this study.

Watson et al*.* reviewed the capabilities for predicting IOL power in patients with KCN. They used actual keratometry for moderate eyes and also some severe eyes. 16 eyes out of 40 moderate eyes were placed within ± 1D from the target refraction. The number of severe eyes in this range has not been reported^6^. Kamiya et al*.* estimated corneal refractive power in the region of 2.4 mm based on the standard keratometry index of 1.3375. When this power was entered into different computational formulas, 8 eyes out of 25 moderate eyes and 0 eyes out of 7 severe eyes were within ± 1D^11^. Savini et al*.* also used the resulted keratometry from different optical biometry devices measurements and stated that, for different IOL power calculation formulas, 1 to 6 eyes out of 13 eyes of stage II and 0 eyes out of 7 eyes of stage III were in the range of ± 1D. It should be mentioned that stage III in this article has been described eyes with an average keratometry of over 53 diopters and those with keratometry values over 55 diopters have not been included in the study^13^.

The achievements from previous studies^6,11,13,15^ show that the actual keratometry values usually result in overestimation of corneal power, which leads to underestimation in IOL power and finally terminate with high hyperopic shift especially in eyes with severe and posterior KCN. It can be said that one of the most important causes of incorrect IOL calculation in these eyes is that this process is usually done by measuring the anterior part of the cornea and estimation of total corneal refractive power is based on the ratio between the anterior and posterior parts of the cornea. It is obvious that such a ratio can be changed due to corneal ectasia process^9,13,16^. Rojas et al*.*^9^ calculated the average keratometry index of 30 eyes with KCN, stated that this index was significantly higher in subjects with KCN than normal subjects. It should be noted that the keratometry index of normal people in this study was also 1.3282, which is different from the standard number of 1.3375. The customized KI calculated in our study was in the range of 1.3210 to 1.3307. The calculation of corneal power by the use of customized KI showed that in comparison with using standard KI, this method reduced the value of PE but did not lead to an acceptable level of error reduction in all eyes.

In addition to assessing the customized keratometry index, we have focused on another method to determine the corneal refractive power. In this method, corneal power was calculated by considering both the anterior and posterior parts of the cornea and by implementing the ray tracing technique. We determined corneal power in two different conditions, including customized refractive index and specific corneal refractive index (1.376), in different areas of the cornea including 0.5, 1, 2, 3 and 4 mm distances from the corneas’ apex. The customized refractive index in each of these areas did not reduce the error to an acceptable level despite expectations. But when the calculated power of the ray tracing method with the refractive index of 1.376 in the 4 mm region was entered into the Barrett formula, PE of all six eyes with KCN dropped dramatically in the range of ± 1D. Consequently, the PE of our two severe eyes in our study, meaning the left eye of our second and third subjects, was calculated as 0.81 and − 0.09, respectively. However, in previous studies, entering the resulted keratometry values from the anterior corneal segment measurement in any IOL computational formulas failed to detect the refractive PE of severe eyes within ± 1D^11,13^. According to the results of our study, the error of our first subject’s eye with PMD was the only one that was not similar to other eyes and it did not place within the range of one diopter. Since the toric lens had been implanted for our first subject, we also used the Barrett toric formula in order to assess the prediction of this computational formula in condition of toric lens implantation. Entering the corneal power of the 4 mm region in this formula, led PE to be zero. A study on patients with PMD and cataract eyes, believes that the refractive outcomes of these cases are highly accurate after cataract surgery^17^ and this is because of the paracentral ectasia region in these corneas, which has less effect on the keratometry values of visual axis zone and thus the IOL is calculated with higher accuracy^18^. However, the PMD eye of our study still has a post-operative hyperopic shift, which requires further studies in this area.

In previous studies, suggestions have been made to determine the best formula for calculating the IOL power in patients with keratoconus, but there is no similar methodology in them. Therefore, it is not possible to compare their results and reach a specific rule for power determination. Leccisotti et al*.*^19^ have measured AL in their study by ultrasound, but did not specify whether they used the contact or immersion biometry method. Also, the method used to select the constant values of formulas has not been expressed. Watson et al*.*^6^ have used the optical biometry for the most eyes, the contact ultrasound biometry was used for the rest of the eyes and for severe keratoconus eyes, the standard value of 43.25 D was used for corneal power. Moreover, PE is considered as the difference between the target refraction and the measured postoperative refraction, which in such studies target refraction does not have any meaning. Hashemi et al*.*^20^ have used different methods for determining corneal power in their study, but he did not state whether he used optimum constants or not. Alio et al*.*^18^ have calculated the IOL power of the short-length eye with HofferQ formula and long-length eyes with the SRK/T formula but he did not provide the results of both formulas in all eyes. Kamiya et al*.*^11^ have evaluated various formulas for determining the IOL power. By examining the anterior corneal power, they have reported a high hyperopic shift, especially in advanced KCN eyes and eventually suggested the SRK/T formula as the most correct one. They did not examine the Barrett Universal II formula, which is considered to be the most correct formula in normal eyes^21–23^. Also, in half of the eyes, the effect of the total corneal power in the 3-mm region was evaluated in the formulas, but they did not state the result by dividing the keratoconus staging in order to determine if this condition contributes to predictive error reduction of the advanced eyes or not. By entering the keratometry of anterior corneal segment and total corneal power into the formulas, the PE of 63% and 55% of eyes, dropped into ± 1 D from target refraction, respectively. They have stated that corneal power is causing a negligible but meaningful myopic shift. Savini et al*.*^13^ has evaluated different formulas, even the Barrett formula, but did not assess the effect of the total corneal power by considering the curvature of both anterior and posterior segments of the cornea. In this study, different values of corneal power by applying standard and customized keratometry index and the posterior corneal segment with standard and customized refractive index in five different formulas were evaluated. Moreover, the basis of calculating the PE was determined as the difference between the postoperative sphere and the predicted refraction of each formula. When we set the postoperative SE as the basis, there was no eye for any of the corneal power in any formula within the prediction error range of ± 1 diopter. But by considering the postoperative sphere, the corneal power of 4 mm region and the Barrett formula the best result was achieved. It seems that if the predicted refraction of each formula is considered to be the estimation of sphere value after surgery, by using our proposed methodology it is possible to select an appropriate IOL power and prevent the occurrence of hyperopic shift.

Finally, despite the few numbers of eyes in this study, it can be said that determining corneal power plays an important role in determination of the correct IOL power in keratoconus patients. Ectasia causes significant changes in corneal geometry and these changes are ignored in standard keratometry and paraxial optics. But in ray tracing, both anterior and posterior surfaces, thickness and asphericity play the role and corneal power can be obtained in different areas. It seems that considering the keratometry index of these patients, results in an overestimation of corneal power, but the resulted power from measuring both posterior and anterior corneal surfaces has better outcomes. Another important factor in determining the IOL power is the ELP estimation that each formula somehow tries to predict it. Based on our results from different formulas, it may be possible to say that the Barrett formula uses a more accurate method for estimating ELP. In fact, regarding appropriate corneal power, ELP estimation has been calculated more precise and a more appropriate IOL power has been obtained. More researches are required to assess the impact of corneal power and prove this study’s solution for reaching correct IOL power.

## Methods

Our Study includes patients which their corneas had PMD or KCN and they had been under cataract surgery and IOL implantation between October 2015 and November 2018. Patients who had surgeries such as CXL, Intracranial ring implantation, corneal transplantation, and refractive surgeries were excluded from the study. Seven eyes from five patients entered into the study.

All surgeries were performed using the standard technique of small-incision phacoemulsification without suture. After creating a 5 or 5.25 mm capsulorhexis and phacoemulsification, an IOL is inserted into the capsular bag through the main incision. The inserted IOLs were include: Acrysof IQ SN6AT9 toric (Alcon Laboratories, Inc., Rochester, NY, USA), Envista MX60 (Bausch & Lomb, Inc., Rochester, NY, USA), Ophthalight ML FLEX 2AS (Mehr Davar, Co., Tehran, Iran), Tecnis ZCB00 (Abbott Medical Optics, Santa Ana, CA), PreciSAL 302AC (Millennium Biomedical, Inc., Cal, USA).

At least 3 months after cataract surgery, patients were examined using slit lamp and auto-refractometer (KR.800, Topcon, Tokyo, Japan). Necessary parameters to simulate patients’ eyes were measured by using devices such as tomography (Pentacam HR, Optikgerate GmbH, Wetzlar, Germany), aberrometry (iTrace, Tracey Technologies, Houston, TX, USA), optical biometry (OLMaster700, Carl Zeiss Meditec, Jena, German) and AS-OCT (Casia, SS1000, Tomey, Nagoya, Japan). A trained operator with high precision carried out all measurements. This study was approved by the Institutional Review Board at Tehran Science and Research University and conducted in accordance with the tenets of Declaration of Helsinki. Informed consent was obtained from all participants.

### Eye modeling

By using achieved measurements from biometry, tomography, aberrometry and AS-OCT devices and information of implanted IOL, pseudophakic eye model of each patient was created. Optical analysis was performed on created models with the application of ray tracing method and by minimizing the difference between the aberrations resulted from ray tracing analysis and aberrations obtained from the aberrometry device; we tried to calculate the corneal refractive index as the only missing parameter in each patient's eye model. In the following steps, we explain how the model is built and how the simulation process is optimized.

### Determine the required distances for model

Axial length (AL) was measured by partial coherence interferometry (PCI) optical biometry^24^. PCI has significantly improved the measurement accuracy, so that it can measure AL compared to ultrasound. The first commercialized device in the market with PCI technique was IOLMaster. For an accurate interpretation of AL, we should mention that the output from the IOLMaster is not the actual length of the eye’s optical path. The achieved values from the IOLMaster device against the immersion ultrasound are calibrated by the use of Eq. (1)^25^:

1 $$Ax_{Zeiss} = \left( {\frac{OPL}{{1.3549}} - 1.3033} \right)/0.9571$$

Which the $Ax_{Zeiss}$ is the output from the PCI and $OPL$ is the optical path length measured by the PCI. Therefore, the length of the optical path can be calculated by the Eq. (2):

2 $$OPL = \left( {Ax_{Zeiss} \times 0.9571 + 1.3033} \right) \times 1.3549$$

The Eq. (3) was used to calculate the eye length from the OPL^25^:

3 $$Ax_{true} = \left( {Ax_{Zeiss} \times 0.9571 + 1.3033} \right) \times 1.3549/1.3616$$

To determine the distance between the posterior part of the cornea to the anterior part of the IOL as well as the thickness of the IOL, the AS-OCT device was used. These measured values were used to determine the location of the IOL and its design.

### Customized modeling of patient's cornea

We used the anterior segment imaging system of Pentacam based on Scheimpflug technology. With the help of this technology, the spatial coordinates from multiple points of both anterior and posterior surfaces of the cornea can be obtained. The most reliable data for building the corneal geometrical model is the raw elevation data of the posterior and anterior parts of the cornea that has not been manipulated by any software algorithm in the topography device^26^. Hence, in the first step, the elevation data of the anterior and posterior parts of cornea were extracted from the device in the form of comma-separated values (CSV format). Then, the data was converted to the Cartesian format by using an algorithm, which was written in MATLAB software version 2018 (Mathworks, Natick, USA).

**Figure 1:**
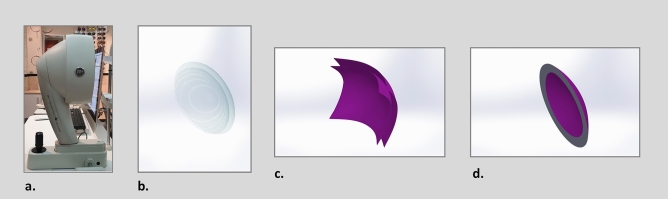
Corneal modeling, (**a**) Pentacam tomography device, (**b**) points cloud data of posterior and anterior parts of the cornea using MATLAB software version 2018a, The MathWorks, Inc., Natick, Massachusetts, United States (https://www.mathworks.com/), (**c**) construction of posterior and anterior surfaces and (**d**) building the solid model of cornea using Solidworks software version 2018, SolidWorks Corp., Waltham MA (https://www.solidworks.com/).

The next step is to produce the posterior and anterior surfaces of the cornea from the geometry of points cloud of the previous step, in which these points cloud, demonstrate the Cartesian coordinates of the points scanned by the topographer in a three-dimensional space. The points cloud of both posterior and anterior parts of the cornea were entered into the SolidWorks software Version 2018 (SolidWorks Corp., MA, USA). By the use of this software, a mesh was generated on the points cloud and the best surface that was fitted into the mesh was selected. To do this, deviation analysis was used to evaluate the difference between the surface and the mesh and based on the smallest difference, the most accurate and possible surface, was created. This process was performed to create both posterior and anterior surfaces. The created surface was then cut to the size of the cornea and finally the gap between the two surfaces was filled and they were knitted together and became like a solid shape. Therefore, with the help of this software, a solid model was built that represents the actual while customized geometry of each patient’s cornea. Figure 1 shows the resulted cornea of the described method. The final model was stored in a format that is compatible with the Zemax software version 2014 (Zemax LLC, WA, USA).

### Patient’s customized IOL modeling

In order to model the implanted IOL for each patient, the specific optical parameters of each IOL provided by the manufacturer companies were used. These parameters included the refractive index and the spherical aberration of the lens and the abbe number (if they exist in the lens specification)^27^. Table 6 shows these specifications for modeled lenses in the study. Since the thickness values of the lenses were not available in the tables provided by manufacturers, the AS-OCT device was used to determine them.

**Table 6:** Optical specifications considered in modeling of the lenses.

Name of IOL	Model	Index of refraction	Abbe number	Induced spherical aberration (μm)
Acrysof IQ	SN6AT9	1.55	37	− 0.2
Envista	MX60	1.54	40.5	Neutral
Ophthalight	ML FLEX 2AS	1.46	–	− 0.25
Tecnis	ZCB00	1.47	55	− 0.27
PreciSAL	302AC	1.5	50	− 0.21

For modeling the lenses, the optical properties of each lens including refractive index, spherical aberration, abbe number, thickness, and refractive power of the lens were used and the radius of anterior, posterior and asphericity conic constant were considered unknown. These unknown parameters were determined by considering the conditions of lens production (i.e., considering the refractive index of the aqueous fluid and vitreous around the lens to achieve the required power) and optimization process in Zemax optical design software. To model Acrysof IQ toric lens, due to the toricity of the posterior side of the lens, the unknown parameters of posterior section consist two parameters including curvature radius of flat and steep axes. After determining the missing parameters, the lens was evaluated for the power and aberration (Table 7).

**Table 7:** Designed lenses in Zemax software (version 2014, Zemax LLC, WA, USA. https://www.zemax.com/) with optimized specifications.

Subjects	S1 (OD)	S2 (OD)	S2 (OS)	S3 (OS)	S4 (OS)	S5 (OD)	S5 (OS)
3D view of designed lenses							
Anterior radius (mm)	17.571	54.575	42.481	500.482	59.104	22.189	64.299
Posterior radius (mm)	− 16.722 (X radius) − 31.442 (Y radius)	12.952	10.901	500.426	− 59.103	− 26.763	− 112.556
Anterior conic constant	− 5.715	0.000	24.404	0.000	− 1.444	− 3.070	− 19.999
Posterior conic constant	0.000	0.000	0.000	0.000	− 1.451	0.000	0.000

### Exact ray tracing and optimization process

In geometrical optics, it is assumed that the wavelength of the light is small enough and the light emission can be described in terms of the beam. The beam path is determined by reflection and refraction, which is defined by the Snell law. Based on Snell law, tracing a large number of beams with finite size in an optical system with defined pupil size is called exact ray tracing or real ray tracing (RRT), which is a well-known approach to analyze the system’s optical characteristics. The use of RRT method leads to a comprehensive description of the optical system, in particular, systems in which there are effects of irregular surfaces. With the help of this approach, any deviations from the ideal optical system can be measured as optical aberrations^27^.

To implement the RRT method, an ophthalmic model must be made firstly. For this purpose, the information obtained from the previous steps, including the cornea (anterior and posterior surfaces), designed IOL and customized interval measurements (including eye length and distance between the cornea and the lens) for each patient were used to build each patient's eye model. In Zemax software, we determined the eyeball using eye length and corneal thickness of the patient. The interior environment of sphere was considered as an environment with refractive index of aqueous humor (1.336^28^). Then the created file of cornea’s surfaces was entered to the software as CAD part, so that all cornea’s measured points were fully entered into the eye model. In the next step, using the pupil center coordinates, pupil size and scan size measured by the iTrace device, we considered the precise location and dimensions of input pupil in the model. After defining the pupil, the individual’s designed IOL was determined in the software by entering its anterior, posterior and its conic constant curvature radii. We determined the vitreous environment in the ocular model by defining an enclosed environment and tangent to the both posterior surface of the IOL and the surface of retina with vitreous refractive index (1.337^28^).

After building a pseudophakic ophthalmic model, it is time to define the aberrations measured by the iTrace device. By studying the order of considering the Zernike coefficients in the iTrace device and checking the standard Zernike coefficients defined in the software, each measured coefficient was compared with its counterpart coefficient in the software. The values of 18 Zernike coefficients from the order of 2 to 5 were entered into the optical design software with the definition of merit function. The coefficients of order 0 and 1, meaning tilt and piston, were eliminated because the piston is only a constant offset and shows absolute distance from the object. The tilt also places the image according to the fovea center, which can be compensated by adjusting the eye relative to the fixation angle^27^. Then we implemented the RRT technique to simulate the process of light propagation and its passage through the optical system of the eye and its arrival at the retina. In this simulation, a wavelength of 785 nm was used to achieve eye aberrations. The optimization approach using Damped Least-Squares method^29^ was used to adapt the aberrations derived from the RRT method with predefined merit functions. In fact, by minimizing the difference between the values of aberrations derived from RRT and the measured ones from the iTrace device, by applying the optimization approach, we tried to achieve value of the refractive index of the cornea as an unknown parameter in the eye model.

### Corneal power measurement

The eyes that were included in the study had Pentacam tomography measurements before cataract surgery. As the method described in the related section of customized corneal modeling of patients, their corneal surfaces were built based on the images taken before surgery and were entered into the optical analysis software. This time, we considered the customized model of patient's cornea that was followed by aqueous humor and with dimensions larger than the individual’s eye length, and the rest of the elements were deleted. Light passes through the cornea and enters to the aqueous humor, after that it goes forward until it focuses in an area and then diverges.

In order to calculate the minimum and maximum corneal power and their corresponding axes, we need to determine the occurrence location of the focusing spot minimum diameter. The minimum and maximum power of the cornea refer to the places that the focus of beams has the maximum and minimum distances from the cornea, respectively. To find these locations, we first determined the location of beam’s focal point in the radial form. In the radial form, the location where the diameter of the spot is minimal in both x and y directions is specified as the focal point. Then we moved the image screen once to 5 mm before this location (to find the maximum power) and once after this location (to find the minimum power). We defined the merit function to find the spot’s smallest diameter and the tilt of cornea around the z-axis considered unknown.

By implementing the Ray Tracing method and optimization process, the cornea was rotated around the Z-axis and the defined merit function was optimized. Thus, the angles for which the smallest diameter of focal spot is placed at the minimum and maximum distance from the cornea, were determined. Then, at the specified angles, we calculated the actual distance from the focus of light to the posterior surface of the cornea. Finally, the refractive index of 1.336 divided to the distance between the focal point and the posterior surface of the cornea, determined the amount of corneal power (Power (D) = 1.336 / f (meters)). Inserting the minimum and maximum distance from the corneal surface as the f value in this formula resulted in calculating the maximum and minimum corneal power.

The described process was repeated in areas with diameters of 0.5, 1, 2, 3 and 4 mm from the cornea, in order to calculate the corneal power at different intervals from the cornea’s apex. In addition, all of the steps mentioned above were performed in two different modes in order to determine the corneal power; once by taking into account 1.376 as the refractive index of the cornea and another time by considering each individual’s eye refractive index obtained from the previous stage.

### Calculating the IOL power

In the previous section, the total power of the cornea was calculated in different regions. In this section, we used the calculated values of corneal power and other preoperative measurements including eye length, anterior chamber depth (ACD) (from epithelium to lens), crystalline-lens thickness, and WTW in order to determine the IOL power. These values were entered into the IOLMaster 700 device to calculate the IOL power by using the SRK/T, Holladay 2, HofferQ, and Haigis formulas. Moreover, the Asia Pacific Association Cataract and Refractive Surgeons website was used for the Barrett Universal II formula v1.05. It should be mentioned that because of the implanted toric lens in the first patient, the Barrett Toric Calculator formula v2.0 was also evaluated for that patient. For the constants of each IOL, the optimized constants were also used which is available on the User Group for Laser Interference Biometry (ULIB) website. For the Ophthalight lens, there were no optimal constants available on this website, therefore we used the constants provided by the manufacturer.

To evaluate the effect of total power of the cornea on the IOL power calculation, the anterior keratometry values that had been measured by the IOLMaster device before surgery were entered into the formulas. These keratometry values were calculated by the use of keratometric index (KI) of 1.3375. Since it has been stated that the values of KI differ in the patients with keratoconus^9^, we decided to also calculate the KI for each eye. According to Eq. (4)^9^, we measured KI by the help of true net power and anterior radius obtained from the Pentacam results before surgery. Then, using the customized KI of each eye, we converted the measured radius values from IOLMaster into keratometry numbers in terms of diopter (Eq. (5)).

4 $$Keratometric\ Index = truenet\ power \times anterior\ radius + 1$$

5 $$K = \left( {KI - 1} \right)/r$$

In formula 5, r is the radius measured in meter and K is the keratometry value in diopter. The keratometry numbers derived from customized KIs are also included in the IOL power calculation formulas.

Patients were examined after at least 3 months from cataract surgery until their refraction riches to a stable condition. Subjective refraction outcome was used to evaluate the optical results in patients. The distance between the patient’s eye and Snellen chart was about 20 feet (6 m). To evaluate each formula, the Prediction Error (PE) was calculated. This error was obtained by subtracting the predicted refraction of each formula (based on the power of implanted IOL for patient) from the actual refraction. The negative PE value indicates that the resulting refraction is more myopic than the predicted refraction and the positive PE value shows a more hyperopic result.

**Figure 2:**
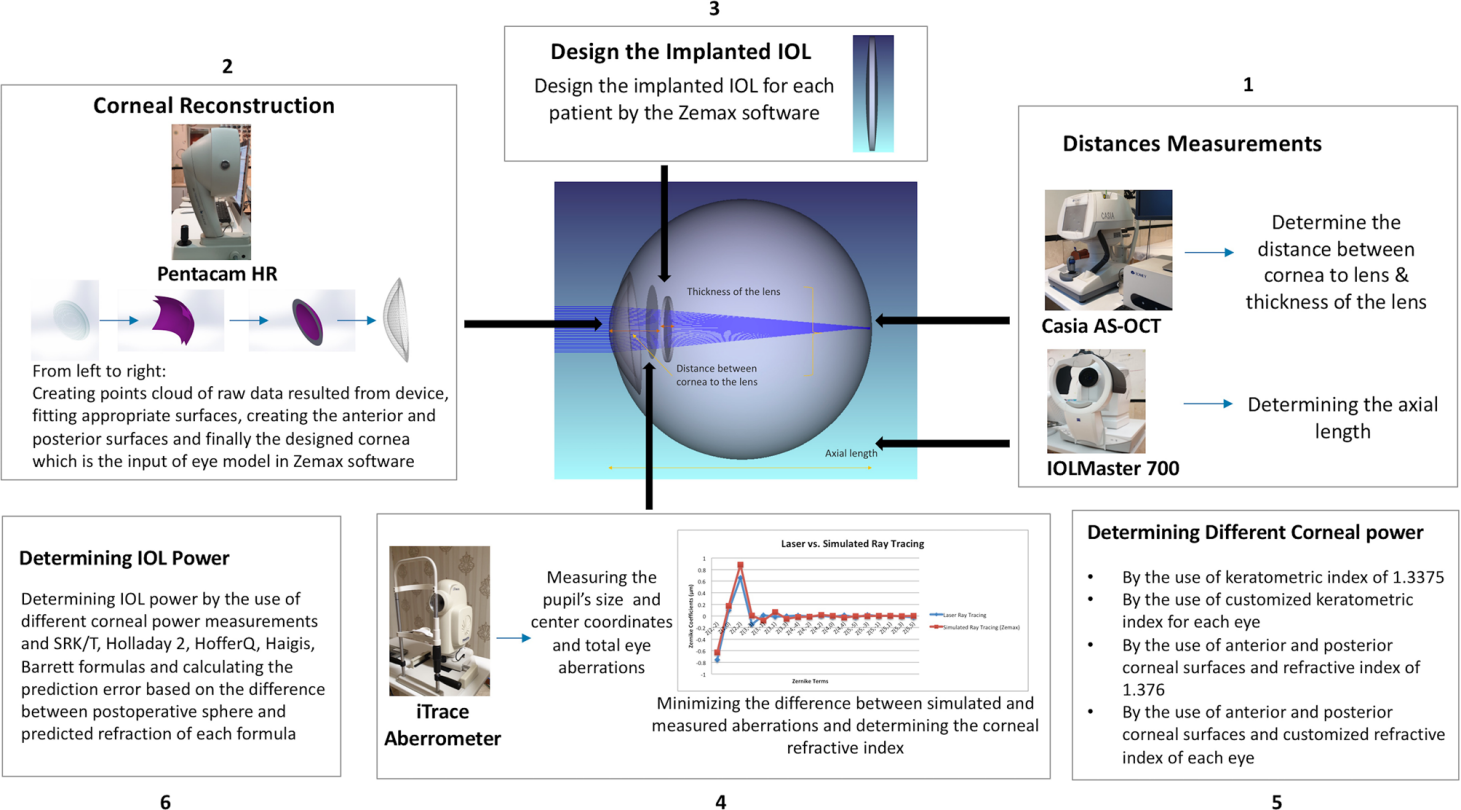
A schematic of different steps in designing the pseudophakic eye model in order to determine the corneal refractive index and also corneal and IOL power calculation by the use of different methods. The middle image in the figure is a customized eye model created using Zemax software (version 2014, Zemax LLC, WA, USA. https://www.zemax.com/).

Figure 2 demonstrates a general overview of implementing our proposed methodology.

## Data Availability

The datasets generated during and/or analyzed during the current study are available from the corresponding author on reasonable request.

## References

[1] Rabinowitz YS (1998). Keratoconus. Surv. Ophthalmol..

[2] Godefrooij DA, De Wit GA, Uiterwaal CS, Imhof SM, Wisse RP (2017). Age-specific incidence and prevalence of keratoconus: a nationwide registration study. Am. J. Ophthalmol..

[3] Jinabhai A, Radhakrishnan H, O’Donnell C (2011). Pellucid corneal marginal degeneration: a review. Contact Lens Anterior Eye.

[4] Moshirfar M, Walker BD, Birdsong OC (2018). Cataract surgery in eyes with keratoconus: a review of the current literature. Curr. Opin. Ophthalmol..

[5] Thebpatiphat N, Hammersmith KM, Rapuano CJ, Ayres BD, Cohen EJ (2007). Cataract surgery in keratoconus. Eye Contact Lens.

[6] Watson MP (2014). Cataract surgery outcome in eyes with keratoconus. Br. J. Ophthalmol..

[7] Antalis JJ, Lembach RG, Carney LG (1993). A comparison of the TMS-1 and the corneal analysis system for the evaluation of abnormal corneas. CLAO J. Off. Publ. Contact Lens Assoc. Ophthalmol..

[8] Olsen T (2007). Calculation of intraocular lens power: a review. ActaOphthalmol. Scand..

[9] de Rojas-Silva M (2014). Keratometricíndex in keratoconic eyes before and after intracorneal ring segment implantation. J. Emmetropia J. Cataract Refract. Corneal Surg..

[10] Shammas HJ (2004). Intraocular Lens Power Calculations.

[11] Kamiya K (2018). Predictability of intraocular lens power calculation for cataract with keratoconus: a multicenter study. Sci. Rep..

[12] Lim DH, Chung T-Y, Chung E-S (2013). Intraocular lens power calculations in a patient with posterior keratoconus. Cornea.

[13] Savini G (2019). Intraocular lens power calculation in eyes with keratoconus. J. Cataract Refract. Surg..

[14] Schallhorn J, Tang M, Li Y, Huang D (2008). Corneal refractive index changes in keratoconus. Invest. Ophthalmol. Vis. Sci..

[15] Piñero DP, Camps VJ, Caravaca-Arens E, Pérez-Cambrodí RJ, Artola A (2014). Estimation of the central corneal power in keratoconus: theoretical and clinical assessment of the error of the keratometric approach. Cornea.

[16] Lisa C (2018). Clinical outcomes of sequential intrastromal corneal ring segments and an extended range of vision intraocular lens implantation in patients with keratoconus and cataract. J. Ophthalmol..

[17] Balestrazzi A (2015). Mini-incision cataract surgery and toric lens implantation for the reduction of high myopic astigmatism in patients with pellucid marginal degeneration. Eye.

[18] Alió JL (2014). MICS with toric intraocular lenses in keratoconus: outcomes and predictability analysis of postoperative refraction. Br. J. Ophthalmol..

[19] Leccisotti A (2006). Refractive lens exchange in keratoconus. J. Cataract Refract. Surg..

[20] Hashemi H, Heidarian S, Seyedian MA, Yekta A, Khabazkhoob M (2015). Evaluation of the results of using toric IOL in the cataract surgery of keratoconus patients. Eye Contact Lens.

[21] Cooke DL, Cooke TL (2016). Prediction accuracy of preinstalled formulas on 2 optical biometers. J. Cataract Refract. Surg..

[22] Kane JX, Van Heerden A, Atik A, Petsoglou C (2016). Intraocular lens power formula accuracy: comparison of 7 formulas. J. Cataract Refract. Surg..

[23] Melles RB, Holladay JT, Chang WJ (2018). Accuracy of intraocular lens calculation formulas. Ophthalmology.

[24] Drexler W (1998). Partial coherence interferometry: a novel approach to biometry in cataract surgery. Am. J. Ophthalmol..

[25] Olsen, T. & Funding, M (2012). Ray-tracing analysis of intraocular lens power in situ. J. Cataract Refract. Surg..

[26] Cavas-Martínez F (2018). Study of morpho-geometric variables to improve the diagnosis in keratoconus with mild visual limitation. Symmetry.

[27] Einighammer J, Oltrup T, Bende T, Jean B (2009). The individual virtual eye: a computer model for advanced intraocular lens calculation. J. Optom..

[28] Malacara-Hernández D, Malacara-Hernández Z (2016). Handbook of Optical Design.

[29] Meiron J (1965). Damped least-squares method for automatic lens design. JOSA.

